# Electrosprayed Ethyl Cellulose Core-Shell Microcapsules for the Encapsulation of Probiotics

**DOI:** 10.3390/pharmaceutics14010007

**Published:** 2021-12-21

**Authors:** Jorge Sevilla Moreno, Panagiota Dima, Ioannis S. Chronakis, Ana C. Mendes

**Affiliations:** DTU-Food, Research Group for Food Production Engineering, Laboratory of Nano-BioScience, Technical University of Denmark, Kemitorvet B202, 2800 Kgs. Lyngby, Denmark; jorgea.smoreno@gmail.com (J.S.M.); pandim@food.dtu.dk (P.D.)

**Keywords:** probiotics, cellulose derivatives, electrohydrodynamics, encapsulation, cell viability

## Abstract

Electrosprayed ethyl cellulose core–shell microcapsules were produced for the encapsulation of probiotic *Bifidobacterium animalis* subsp. *lactis* (Bifido). Ethyl cellulose (ETC) was used as a shell material with different core compounds (concentrated Bifido, Bifido–maltodextrin and Bifido–glycerol). The core–shell microcapsules have an average diameter between 3 µm and 15 µm depending on the core compounds, with a distinct interface that separates the core and the shell structure. The ETC microcapsules displayed relatively low water activity (aw below 0.20) and relatively high values of viable cells (10^9^–10^11^ CFU/g), as counted post-encapsulation. The effect of different core compounds on the stability of probiotics cells over time was also investigated. After four weeks at 30 °C and 40% RH the electrospray encapsulated samples containing Bifido–glycerol in the core showed a loss in viable cells of no more than 3 log loss CFU/g, while the non-encapsulated Bifido lost about 7.57 log CFU/g. Overall, these results suggest that the viability of the Bifido probiotics encapsulated within the core–shell ETC electrosprayed capsules can be extended, despite the fact that the shell matrix was prepared using solvents that typically substantially reduce their viability.

## 1. Introduction

Probiotics are live microorganisms that are intended to have health benefits when consumed or applied to the body. Probiotics are generally sensitive to harsh environmental and processing conditions such as low pH, digestive enzymes, heat treatment, water activity, and molecular oxygen. To ensure probiotics’ healthy benefits, their metabolic activity has to be preserved in foods and during the complex digestive process until they reach the target site of the gastrointestinal tract (GIT) [1,2].

Probiotics’ health benefits can be ensured by supplying a minimum of 10^6^–10^8^ CFU of live probiotics per gram of foodstuff [3]. Thus, probiotics need to be protected from the adverse conditions they are exposed to during food processing, storage, and the gastrointestinal digestion process. Encapsulation offers the possibility to preserve probiotics´ viability from several harmful factors, and ensure their release at targeted sites in the digestive tract [4].

Several technologies are available for the encapsulation of probiotics in foods (e.g., spray-drying, spray-cooling, fluid-bed coating, extrusion, and complex coacervation followed by drying) [5]. Among all the main technologies, spray- and freeze-drying are the most common processes used in the food industry for the microencapsulation of active ingredients [5] including probiotics [2]. However, those technologies expose the probiotic cultures to harmful temperatures and pressures, which consequently cause a considerable reduction in bacterial viability [2].

Alternatively, electrospray stands out as a promising encapsulation technology of probiotics, producing dried nano-microcapsules from the breakup of a flowing liquid by means of an electrical field, at room temperature without heat, and without affecting the viability of the living cells [1]. Furthermore, it allows relatively high encapsulation efficiency [6], in addition to the possibility of using a broad range of capsule ingredients [7]. The functionality of electrosprayed capsules can be enhanced by the co-processing of biopolymer mixtures and by tuning their surface properties (e.g., crosslinking) [7] or by the utilization of co-axial electrospray methods to create core–shell structures [5].

Polysaccharides such as pullulan [8], alginate [6,9], starch-based ingredients [6,10], maltodextrin [11], and acacia gum [2] have been utilized as electrospray matrices to encapsulate probiotic cells with high encapsulation and stability efficiency.

For instance, *Bifidobacterium animalis subsp. lactis* Bb12 was encapsulated within electrosprayed capsules consisting of whey protein concentrate (WPC) and pullulan [8]. *Lactobacillus acidophilus* cells were encapsulated within electrosprayed core–shell alginate–zein microcapsules [9]. In another study, WPC was also used to encapsulate *Lactobacillus plantarum* [12] and freeze-dried *Bifidobacterium longum subsp. infantis* CECT 4552 [11].

Ethyl cellulose (ETC) is a linear polysaccharide produced by the replacement of the cellulose’s hydroxyl end groups with ethyl end groups. ETC is non-water soluble and more hydrophobic than other commercial cellulose ethers [12], and it has been proved to be physiological inert, tasteless, odorless, non-caloric [13], non-toxic, and stable during storage [14,15], which makes it suitable for the encapsulation, protection, and delivery of bioactive compounds [14,16]. Commercially, ETC has been used in formulations conceived for oral and topical pharmaceutical applications including drug delivery and as a coating agent for tablets and granules to control the drug-release rate at the GIT track [12,15,17].

Furthermore, it has been demonstrated that ETC can be used to produce electrospun fibers and electrosprayed capsules [14,16,18,19,20,21,22]. For instance, ketoprofen was encapsulated within electrosprayed ETC microparticles [14]. ETC with model hydrophilic components (vanillin, ethylmaltol, and maltol) was also included as core material in electrosprayed core–shell capsules, using stearic acid as a model lipid monolayer at the shell [16]. However, ETC has never been studied for the encapsulation of probiotic cells.

Based on the potential of ETC to be used as encapsulating material for bioactives, in this study electrosprayed core–shell ETC microcapsules were developed for the encapsulation and protection of *Bifidobacterium animalis* subsp. *lactis* (Bifido) probiotic cells. Different compounds (concentrated Bifido cells, Bifido–maltodextrin and Bifido–glycerol) were included within the microcapsule core. ETC microcapsules’ functionality to preserve the viability of the encapsulated Bifido probiotics was evaluated.

## 2. Materials and Methods

### 2.1. Materials

All chemicals and materials used in this study, including ethyl cellulose (48.0–49.5% (*w*/*w*) ethoxy groups, degree of substitution of 2.5 [17], viscosity 30–70 mPa.s, 5% in toluene/ethanol 80:20 (25 °C)), and glycerol were acquired from Sigma-Aldrich (Steinheim, Germany) unless otherwise indicated. The chemicals were used as received. Maltodextrin, with a commercial name of GLUCIDEX® 12 (dextrose equivalent of 12, Mw of 23 kDa [23]) was obtained from Roquette (Lestrem, France).

### 2.2. Bacteria Culture

*Bifidobacterium animalis* subsp. *lactis* (Bifido) (strain deposit number DSM 33443) was selected as the model strain supplied by Chr Hansen A/S (Hørsholm, Denmark). The strain was subjected to freeze pelletization, and stored at −80 °C.

### 2.3. Electrospray Solutions Preparation

The electrospray shell solution was prepared by dissolving 8% *w*/*v* of ETC in ethanol 96% (VWR International, Darmstadt, Germany) or 8% *w*/*v* of ETC in acetone. The core solutions were prepared after thawing the Bifido cells at ambient conditions and mixing them with the different compounds (Table 1). For S2, 22.5 mL of thawed Bifido was gently added to a maltodextrin solution prepared by dissolving 30 g of maltodextrin in 35 mL of Mili-Q water. The core solutions of S3 and S4 were prepared by adding 30 mL of thawed Bifido mixed with 13 mL of glycerol.

### 2.4. Co-Axial Electrospray of ETC Core–Shell Capsules Encapsulating Bifido

The electrospray setup included: (i) two syringe pumps (New Era Pump Systems, Inc., Farmingdale, NY, USA), each one feeding the shell and the core solution at the flow rate of 0.42 mL/min and 0.21mL/min to the co-axial needle, respectively (concentric needles with an inner diameter of 1.42 mm for shell solution and 0.48 mm for core solution), and (ii) a high voltage generator (ES50P-10W, Gamma High Voltage Research, Inc., Ormond Beach, FL, USA) was utilized to provide an applied voltage of +30 kV at the co-axial needle and an applied voltage of −5 kV at the collector. The distance from the co-axial needle to the collector was 10 cm. The electrospraying process was performed in a fume hood to keep the process under 30% relative humidity using nitrogen. The collected capsules were them removed from the collector and stored at −80 °C prior further analysis.

### 2.5. Microscopy Analysis

The size and morphology of the electrosprayed ETC core–shell capsules and free Bifido were investigated using a scanning electron microscope (SEM). Free Bifido samples were fixed with glutaraldehyde (Electron Microscopy Sciences, Hatfield, PA, USA) solution (2.5%, *v*/*v* in 0.1 M phosphate-buffered solution, pH 7.4) for 1 h. The samples were then dehydrated in a graded ethanol series (50, 70, 90, 96, and 100%) followed by immersion in hexamethyldisilazane (HMDS). Samples (free Bifido and ETC core–shell capsules loaded with Bifido) were coated with 6 nm of gold for better conductivity using a sputter coater (Leica Coater ACE 200, Leica, Vienna, Austria) and imaged in a Quanta FEG 3D SEM (FEI, Eindhoven, Netherlands). The microcapsule diameter was calculated using Image J analysis software (National Institutes of Health, Bethesda, MD, USA), by measuring 50 different capsule diameters for each sample. The average diameter was calculated from these measurements for each microcapsule.

To evaluate probiotics distribution inside the capsules, the microcapsules with encapsulated Bifido cells were dispersed in NaCl 0.9%. Incubation with RedDot (Biotium, Fremont, CA, USA) at 37 °C for 10 min protected from light was performed prior to visualization using an Axioplan Imager Z1m microscope (Zeiss, Oberkochen, Germany).

### 2.6. Fourier Transform Infrared Spectroscopy (FT-IR)

FTIR spectra of the core–shell microcapsules loading Bifido and free Bifido cells were obtained using a FTIR spectrometer (Spectrum 100, FT-IR spectrometer, Perkin Elmer, Waltham, MA, USA). The FTIR spectra were acquired at a wavelength ranging from 4.000 cm^−1^ to 650 cm^−1^ at room temperature (20 ± 2 °C). Each measurement was derived an average from 4 scans.

### 2.7. Water Activity (aw)

The water activity (aw) of the ETC–Bifido microcapsules was measured using a water activity analyzer (LabMaster-aw basic, Novasina, Lachen, Switzerland), at 25 °C.

### 2.8. Cell Viability, Encapsulation Efficiency, and Storage Stability

Electrosprayed samples containing *Bifidobacterium animalis* were stored in a desiccant cabinet, (NALGENE^®^ 5317-0120, Thermo Fisher Scientific™, Rochester, NY, USA), containing a saturated solution of potassium carbonate at a relative humidity (RH) of 40% at 30 °C. The viability of encapsulated and non-encapsulated Bifido cells was determined by colony-forming unit (CFU) analysis. The samples were diluted 1:100 with Maximum Recovery Diluent (MRD, (Oxoid CM0733, Hampshire, UK)) at 37 °C.

The suspension was homogenized by stomaching (Smasher, AES Laboratoire, bioMérieux, Hazelwood, MO, USA) at intermediate speed for 2 min, and further dilutions were performed in MRD. Appropriate volumes of appropriate dilutions were plated in de Man Rogosa Sharpe (MRS) agar supplemented with 0.05 *w*/*v* % L-cysteine hydrochloride monohydrate by pour plating, aiming for colony counts between 30–300 CFU/plate. Plates were incubated at 37 °C under anaerobic conditions for 3 days, and the results were calculated as the average of 2 plates. Cell viability was measured at the time points of 0, 2, and 4 weeks.

The encapsulation efficiency (EE) of the microcapsules was calculated by determining the number of viable cells inside the capsules (N) divided by the number of viable cells in the initial solution (N0), as expressed in (1) [2]:

(1) $$\mathrm{EE}\;\left(\%\right)=\frac{\mathrm{Log}\;N}{\mathrm{Log}\;N0}\times 100$$

### 2.9. Data Analysis

Student’s *t*-test was applied to determine statistical significance using Excel software. One-tailed unpaired t-test with 95% confidence interval was considered statistically significant if *p* < 0.05 (*).

## 3. Results and Discussion

### 3.1. Microcapsules and Cells Size and Morphology

**Figure 1:**
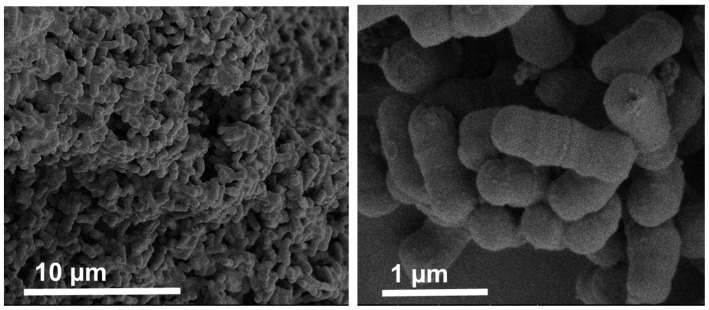
SEM images of free Bifido cells at different magnifications.

The Bifido cells used in this study displayed a typical rod-like structure, with an average length of about 1–1.5 µm as observed by SEM (Figure 1), which is in agreement with previous studies [24].

**Figure 2:**
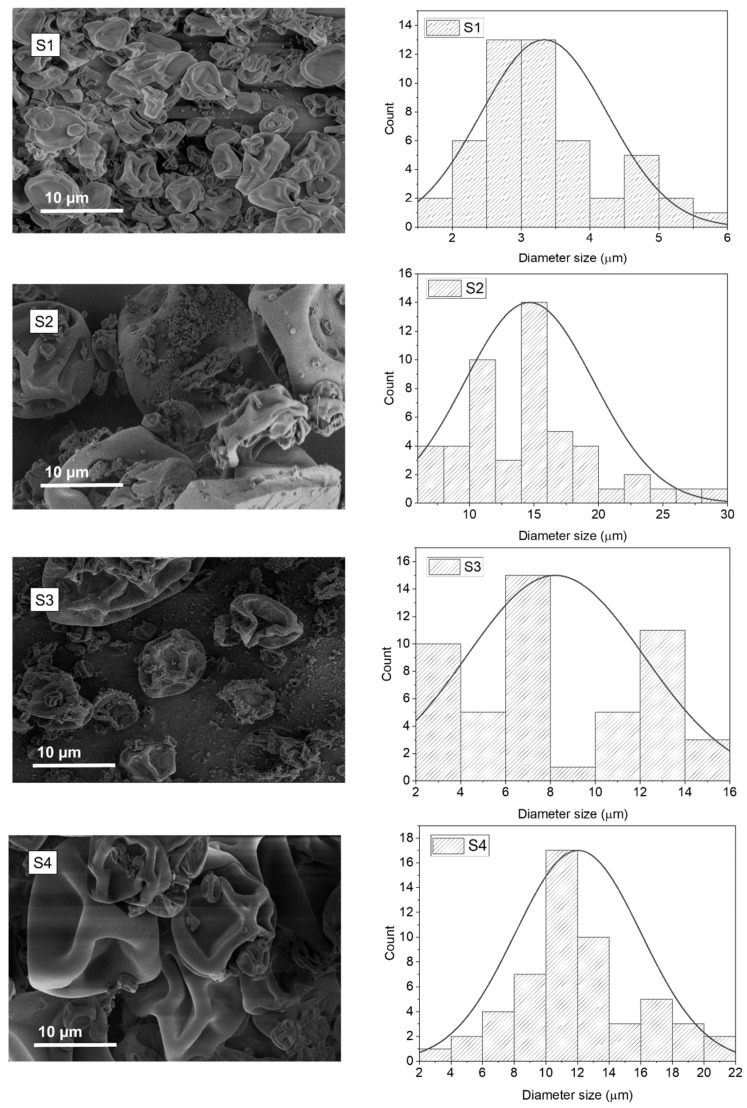
SEM images of ETC core–shell capsules loading Bifido (samples **S1**–**S4**) and respective histograms displaying microcapsules diameter distribution.

The size and the morphology of the electrosprayed ETC core–shell capsules loaded with Bifido are shown in Figure 2. Overall, the core–shell microcapsules revealed a dimpled structure morphology, which resembles the morphology of electrosprayed ETC-based capsules [14]. The lack of spherical structure of these particles could be related with the fast evaporation of the solvent, as both ethanol and acetone are highly volatile solvents. Electrosprayed capsules with smoother and spherical-like surfaces can be obtained using solvents with a lower evaporation rate [25].

The electrosprayed core–shell capsules that contain only Bifido in the core (S1), displayed an average diameter of 3.33 ± 1.18 µm (Table 2). However, when maltodextrin (S2) and glycerol (S3) were incorporated in the microcapsule core, the average diameter of the capsules was increased to 14.63 ± 4.99 and 8.21 ± 3.96 µm, respectively (Table 2). This could be due to the increase in the viscosity of the core solution.

An increase in the average diameter of the microcapsules was also observed when acetone was used as ETC solvent (S4), in comparison to the capsules prepared using ethanol (S3) (Figure 2 and Table 2). Furthermore, the higher average diameter determined for S4 comparatively to S3 could be probably due to the higher polydispersity of the microcapsules size produced with ethanol (S3) (Table 2) and due to the higher encapsulation efficiency of cells in sample S4.

**Figure 3:**
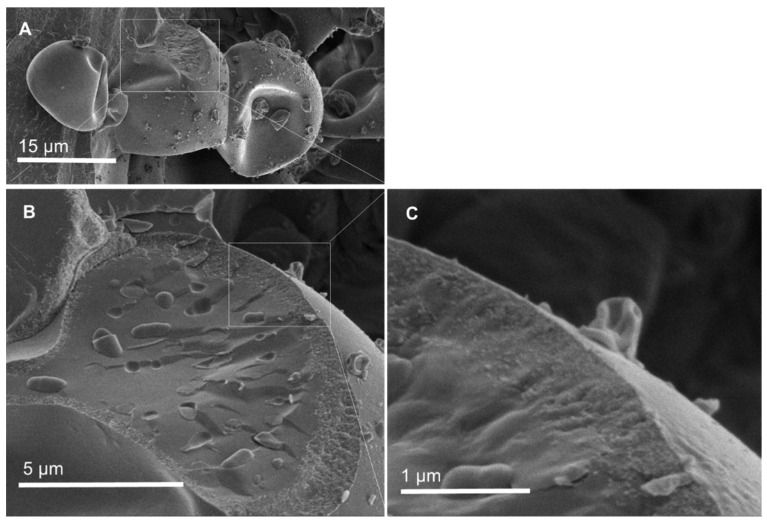
SEM images showing the overall structure of electrosprayed ETC core–shell microcapsules loading Bifido: (**A**) the microcapsule cross-section with (**B**) the distinct core and shell materials, and the Bifido cells distributed within the core of the microcapsules; (**C**) the interface between ETC (shell) and glycerol (core).

Figure 3 shows a cross-section of core–shell electrosprayed ETC capsules prepared with acetone as the shell solvent (S4). A distinct interface that separates the core of the capsule (containing the Bifido cells imbedded in glycerol) from the ETC shell can be observed. Thus, it is presumed that fast outer shell solvent evaporation (in this case acetone) and subsequent shell solidification prevents the diffusion and mixing of the core and shell compounds. A distinct interface between the core and the shell was also noticed in a previous study of core–shell electrospun starch fibers for the encapsulation of probiotics [10].

**Figure 4:**
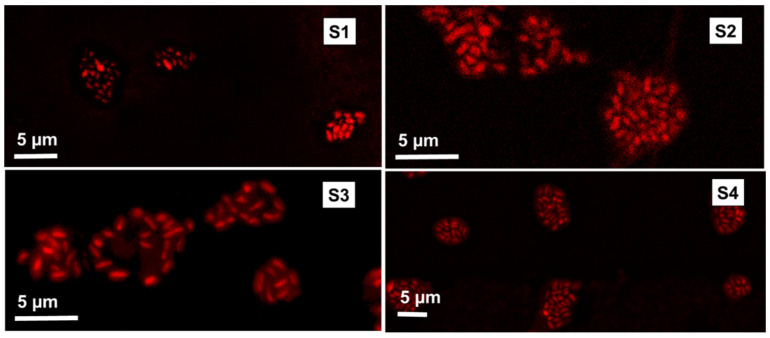
Confocal microscopy images of the Bifido encapsulated within the electrosprayed ETC core–shell microcapsules.

To study the distribution of the encapsulated cells within the microcapsules, the cells were stained and analyzed using confocal microscopy. Figure 4 confirms that the Bifido cells were effectively encapsulated and homogeneously distributed within the different ETC core–shell microcapsules.

### 3.2. Water Activity (aw)

The composition of the capsule, the permeability of oxygen, and the aw are key parameters to preserve the viability of probiotic cells [3]. In respect to the aw, the optimal survival of probiotic cells occurs at aw < 0.3 [26], where probiotics can maintain their functionality after drying. The aw of the electrosprayed ETC–Bifido microcapsules ranged from 0.13 to 0.20 (Table 2).

**Figure 5:**
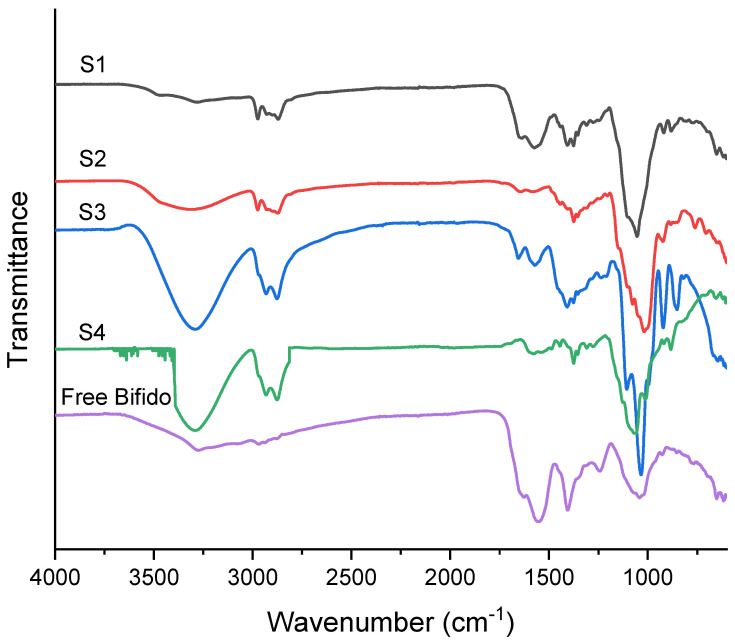
FTIR spectra of Bifido cells encapsulated within electrosprayed ETC core-shell microcapsules, and non-encapsulated (free) Bifido.

When ethanol was used as the ETC solvent for the microcapsule shell, the water activity increased in the order S1 < S2 < S3 (not statistically significant). The slightly higher water activity of the sample S2 comparatively to S1 could be attributed to the presence of hydrophilic maltodextrin, which increases the affinity for water binding. The highest value of aw was found for S3, most likely due to the hydrogen bonding and molecular dynamics of the water of the aqueous dispersion of Bifido–glycerol mixtures in the core [27], which is in agreement with FTIR results (Figure 5).

### 3.3. FTIR

The FTIR spectra of free Bifido (Figure 5) shows three distinct bands at 3208, 1629, and 1550 cm^−1^ corresponding to amide groups of the existing proteins at the cell membrane. Furthermore, the bands at 2968, 2973, and 2878 cm^−1^ are mainly attributed to the asymmetric stretches of methyl and methylene groups of the lipid components of the membrane, mainly fatty acids, as also suggested elsewhere [28,29]. Furthermore, a band at 1401 cm^−1^ could be related to the (C-O) vibrations of COO- groups from esterified lipids and fatty acids [28]. The bands assigned at 1244 and 1043 cm^−1^ are provided by the (P=O)s of PO^2−^ groups [30].

The shell compound of the microcapsules was ethyl cellulose, and consequently a distinct peak at 3464 cm^−1^ was observed, due to the -OH groups of the polysaccharide backbone [14]. The intra- and intermolecular hydrogen bonding due to the -OH groups is more intense for the samples containing glycerol (S3 and S4). The asymmetric peaks of -CH stretching can be seen in the peak regions of 2970–2870 cm^−1^. Other relevant vibration peaks around 1054–1011 and 1275 cm^−1^ were mainly attributed to C-O-C stretch and C-H bending, respectively [30]. It is important to note that the C-O-C stretch does not display the same absorption wavenumber, probably due to changes in the bond strength and force constant of C-O-C bond related with the different solvents.

### 3.4. Cell Viability and Stability over Time

**Figure 6:**
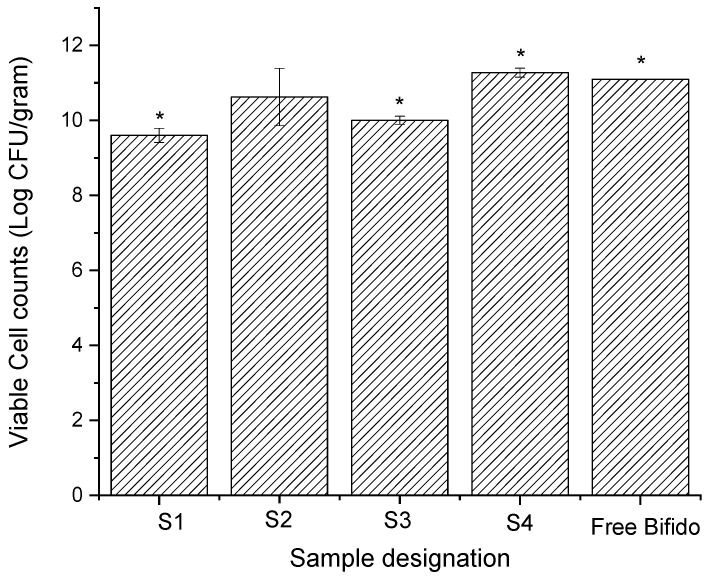
Viable cell counts post-encapsulation of Bifido probiotic cells encapsulated within electrosprayed ETC core-shell microcapsules and non-encapsulated (free) Bifido. All values are mean ± standard deviation, and * indicates a significant difference (*p* < 0.05).

Figure 6 displays the number of viable cells determined by the plate counting method post-encapsulation. Furthermore, free Bifido viable cells (non-encapsulated) were also determined and displayed an initial cell count of 11.10 log CFU/g. The encapsulation efficiency (EE) determined for viable cells encapsulated within ETC electrosprayed microcapsules was about 39.6, 44.8, 77.6, and 87.41 %, for S1, S2, S3, and S4, respectively (Table 2).

For the samples prepared using ethanol as a shell solvent (S1–S3), S1 displayed the lowest value of viable probiotics at 9.62 log CFU/g. The number of viable encapsulated probiotics for S2 and S3 was about 10.90 log CFU/g and 10.01 log CFU/g, respectively. The increase in the initial viable cells counts of the sample S2 in comparison to S1 suggests that maltodextrin has some protective effect on Bifido (note the similar encapsulation efficiency of these samples). Sample S4 (ETC shell electrosprayed using acetone), showed slightly higher cell viability counts (11.28 log CFU/g) in respect to S3 (ETC shell electrosprayed using ethanol). The higher number of viable encapsulated probiotics detected in S3 and S4, in respect to S1, could be due to the higher initial cells number, the higher EE, and also due to the protection of the glycerol core compound. Overall, these results suggest that the probiotic cells encapsulated within the core-shell ETC electrosprayed capsules preserved their viability after electrospraying, despite the fact that the shell matrix was prepared using solvents that typically substantially reduce their viability. This further confirms that the solvent of the shell matrix (ETC) did not migrate into the core of the microcapsules during electrospray processing, where the probiotics were mostly located (Figure 3).

**Figure 7:**
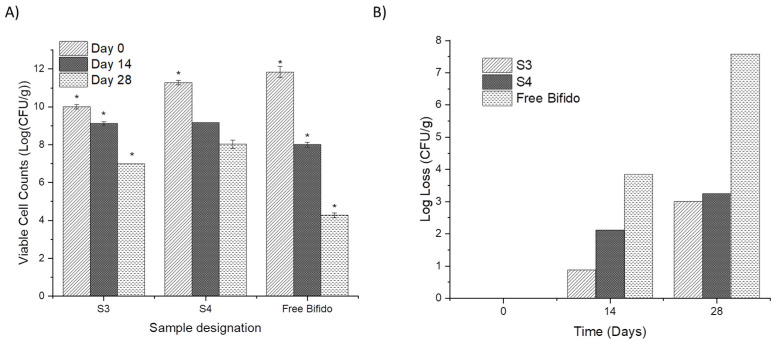
Viable cell counts (**A**) and log loss of viability (**B**) over time (30 °C and 40% RH) of encapsulated Bifido within electrosprayed ETC core-shell microcapsules, and non-encapsulated (free) cells. All values are mean ± standard deviation, and * indicates significant difference (*p* < 0.05).

Due to the higher encapsulation efficiency obtained for samples S3 and S4, their stability over time was also assessed using plate counting (30 °C and 40% RH) and compared with non-encapsulated Bifido cells (free Bifido) (Figure 7). In the first two weeks of the stability study, a reduction in the viable cells from 10.01 log CFU/g to 9.13 log CFU/g was observed for S3. Cells encapsulated in S4 showed a reduction from 11.28 to 9.16 log CFU/g.

The non-encapsulated Bifido cells lost about 3.84 log CFU/g of their viability, within two weeks, and showed a log loss of 7.57 CFU/g after four weeks at 30 °C and 40% RH. The electrospray encapsulated Bifido cells only lost about 3 log CFU/g of viable cells (Figure 7B). Previous studies also suggested that glycerol has a protective effect on probiotics’ stability [10,31,32].

## 4. Conclusions

Electrosprayed ethyl-cellulose core–shell microcapsules were produced for the encapsulation of Bifido probiotic cells. The average diameter of the core-shell capsules was between 3 µm and approximately 15. A distinct shell and core microstructure were observed, indicating that the shell matrix of ETC and the solvent did not migrate into the core of the capsule, where the probiotics were mostly located. The core–shell microcapsules of ETC displayed a relatively low aw (below 0.20) and relatively high values of viable cells (10^9^–10^11^ CFU/g) counted post-encapsulation. After four weeks at 30 °C and 40% RH, the encapsulated samples contained Bifido–glycerol in the core, showed a loss in viable cells no more than 3 log CFU/g, while the non-encapsulated Bifido lost about 7.57 log CFU/g. Overall, the present study clearly demonstrates the potential of electrosprayed ETC core–shell microcapsules to encapsulate probiotic cells and to extend their viability over time.

## Figures and Tables

**Table 1:** Sample designation for the different core–shell microcapsules made of ethyl cellulose in the shell.

Sample Designation	Solvent in Shell	Core Composition
S1	Ethanol	Bifido
S2	Ethanol	Bifido–maltodextrin
S3	Ethanol	Bifido–glycerol
S4	Acetone	Bifido-–glycerol

**Table 2:** Average diameter, water activity (aw), and encapsulation efficiency (EE) of the electrosprayed ETC core–shell microcapsules loading Bifido cells.

Sample Designation	Diameter Size /µm	PDI	aw	EE%
S1	3.33 ± 1.18 *	0.083	0.13 ± 0.028	39.6
S2	14.63 ± 4.99 *	0.12	0.16 ± 0.010	44.8
S3	8.21 ± 3.96 *	0.23	0.20 ± 0.029	77.6
S4	12.04 ± 3.98 *	0.09	0.18 ± 0.066	87.4

* All values are mean ± standard deviation. * indicates significant difference (*p* < 0.05).

## Data Availability

Not applicable.
